# Sex differences in management and outcomes of patients with atrial fibrillation in the Middle East: Gulf survey of atrial fibrillation events (Gulf SAFE)

**DOI:** 10.1371/journal.pone.0175405

**Published:** 2017-05-17

**Authors:** Abdulla Shehab, Mohammad Zubaid, Akshaya Srikanth Bhagavathula, Wafa A. Rashed, Alawi A. Alsheikh-Ali, Wal AlMahmeed, Kadhim Sulaiman, Ibrahim Al-Zakwani, Ahmed AlQudaimi, Nidal Asaad, Haitham Amin, on behalf of the Gulf Survey of Atrial Fibrillation Events (Gulf SAFE) investigators

**Affiliations:** 1 Department of Internal Medicine, Faculty of Medicine, UAE University, Al Ain, United Arab Emirates; 2 Department of Medicine, Faculty of Medicine, Kuwait University, Kuwait City, Kuwait; 3 Department of Clinical Pharmacy, University of Gondar-College of Medicine and Health Sciences, Gondar, Ethiopia; 4 Department of Medicine, Mubarak Al-Kabeer Hospital, Ministry of Health, Kuwait University, Kuwait City, Kuwait; 5 Division of Cardiology, Sheikh Khalifa Medical City, Abu Dhabi, United Arab Emirates; 6 Royal Hospital, Muscat, Oman; 7 Department of Pharmacology and Clinical Pharmacy, College of Medicine and Health Sciences, Sultan Qaboos University and Gulf Health Research, Muscat, Oman; 8 Al Thawra Hospital, Sana’a, Yemen; 9 Department of Cardiology and Cardiovascular Surgery, Hamad Medical Corporation, Doha, Qatar; 10 Mohammed Bin Khalifa Cardiac Center, Manama, Bahrain; Indiana University, UNITED STATES

## Abstract

Differences in the management of atrial fibrillation (AF) between men and women were investigated by using Gulf SAFE data in the Middle East. The study included 2,043 patients presenting with AF to emergency room (ER) were prospectively enrolled and followed for one-year. Women were older, have higher body mass index (BMI), comorbidities, and health complications than men. With regard to management of AF, cardioversion was recommended more often for men (16.7% vs. 9.3%), and underwent electrical cardioversion (2.2% vs. 1.1%). Women were prescribed digoxin more frequently than men (25.6% vs. 17.4%) and a significant number women received warfarin alone (31.1% vs. 8.7%). No difference between the sexes was noticed in One-year rates of stroke/transient ischemic attacks (TIA) and all-cause of mortality after one-year follow-up (3.1% men vs. 3.3% women, and 7.5% vs. 7.4%). Older age (≥ 65 years), smoking, alcohol use, CHADS2 scores ≥5 were some of the significant risk factors in men with AF. Suboptimal use of anticoagulants, higher mortality and stroke/TIA events at one year are high but similar between the sexes. ER management revealed high use of rate control strategy and high rate of hospital admission was noticed in women.

## Introduction

Atrial fibrillation (AF) is the most common chronic arrhythmia affecting approximately 9% of population and is treated according to standard guidelines [1]. The most serious consequences of AF is the propensity for thrombus formation due to stasis in the atria causing embolization and devastating the cerebral circulation. During the past century, a range of contributing risk factors were identified in patients with AF including female-gender for stroke [2]. However, sex-specific differences between men and women remains controversial. For instance, a national wide cohort study conducted on Swedish female population with AF did not identify female sex as an independent risk factor for stroke [2]. Similarly, J-RHYTHM registry study on Japanese AF population did not confirm the higher stroke risk in females [3]. In contradictory, meta-analysis by *Madias and Trohman*, and *Wagstaff et al*. reported a higher stroke risk among female patients with AF [4,5]. Indeed, the incidence and prevalence of AF was much lower in Asia-Pacific region compared to Western countries and no differences between the sexes was noticed [6,7]. Findings from Framingham Heart Study showed sex-specific AF risk-factor-adjusted-odds for death is slightly higher in females than males (1.9 vs. 1.5) [2]. These discrepancies reflects failure of the ‘one size fits all’ approach. A better understanding of the sex difference could be helpful to improve management and risk reduction in patients with AF. Therefore, we aimed to investigate the management and outcomes in relation to gender among AF patients in Gulf region of the Middle East.

## Methods

Gulf Survey of Atrial Fibrillation Events (Gulf SAFE) is a prospective observational cohort registry of consecutive adult (≥18 years) patients with AF presenting to emergency room (ER) of 23 hospitals in six Gulf countries of the Middle East, between October 2009 and June 2010, and were followed prospectively for one year after enrollment. Full details of study design of the Gulf SAFE registry have been previously described in detail [8]. The eligible criteria includes adult patients, having AF >30 seconds on a 12-lead electrocardiogram (ECG) or rhythm strip while in ER, and willing to provide written informed consent. All the patients with AF were enrolled regardless of the primary reason for their ER visit and management was based on treating physician discretion. Patients were followed at 1, 6 and 12 months from the time of enrollment while attending clinic or telephonic interview. Data of the Patients with AF having rheumatic mitral stenosis or a prosthetic heart valve (i.e. non-valvular AF) were not included in the analysis.

### Ethical considerations

The study protocol was approved by the ethical committees of 25 institutions/ hospitals in 6 Middle East countries: To state some Al Tawaa hospital- Yemen, Royal hospital- Oman, Ministry of Health- Kuwait, Al Qassimi Hospital, Dubai Health Authority, Al Ain hospital and Alnoor hospital—United Arab Emirates, Hamad Medical Corporation- Qatar, and Mohammed Bin Khalifa Bin Salman Al Khalifa Cardiac center- Bahrain. We strictly followed 1975 Declaration of Helsinki ethical guidelines. Written informed consent was obtained from each patient prior to their enrollment in the study. We used standard case report form (CRF) and entered the data online. Data characteristics and outcome variables were similar to that of previous published Gulf SAFE study [9]. The full list of Gulf SAFE committee members, national coordinators and investigators were attached in supporting information file (S1 File).

### Statistical analysis

All the statistical analyses were performed using SPSS version 21.0. Frequencies and percentages were used for the discrete variables (χ^2^ test or Fisher’s exact test) and mean with standard deviations (SD) for continuous variables. Multivariate logistic regression was conducted to identify independent risk factors associated with the outcomes between sexes. Model fit of the regressions were analyzed using Hosmer & Lemeshow statistics and area under the receiving operating curve (ROC). A *P*-value of <.05 was considered as a cutoff value for statistical significance.

## Results

The Gulf SAFE enrolled 2043 patients (1063 men and 980 women) with AF patients. Of these, majority (84%; 956 men and 764 women) had non-valvular AF. The women were slightly older (58.5±16.2 years) than men (55.1±16.5 years) *p*<0.001 had higher BMI (28.6±7.2 kg/m^2^ vs. 27.3±5.3 kg/m^2^; *p*<0.001), and comorbid conditions such as hypertension (58.1% vs. 47.3%), hyperlipidemia (36.3% vs. 30.4%), diabetes mellitus (32.3% vs. 27.1%), rheumatic heart disease (21.5% vs. 10%), valvular heart disease (21% vs 17.6%) and showed higher prevalence of TIA attack (4.9% vs. 2.8%; *p*<0.001). In addition, a higher number of women also had permanent AF (28.1% vs. 26.9%) and CHADS_2_ scores (1.6±1.3 vs. 1.3±1.3). The most common reasons for ER presentation in men was AF (48.5% vs. 41.5%) and non-cardiac in women (30.2% vs. 23.1%) (Table 1).

**Table 1:** Baseline characteristics of study patients with non-valvular atrial fibrillation*.

	Men (n = 1063)	Women (n = 980)	Total (n = 2043)	*P*
**Age (years)**	55.1±16.5	58.5±16.2	56.7±16.4	NS
**Age ≥65 years**	325 (30.9)	390 (39.8)	718 (35.1)	<.001
**Body mass index**^†^ **(kg/m**^**2**^**)**	27.3±5.3	28.6±7.2	27.9±6.3	<.001
Underweight, <18.5	19 (1.8)	31 (3.4)	50 (2.6)	
Normal weight, 18.5–24.9	331 (32.8)	285 (31.1)	616 (32)	
Overweight, 25–30	422 (41.8)	264 (28.8)	686 (35.7)	
Obese, >30	236 (23.4)	334 (36.5)	570 (27.6)	
LA diameter^‡^, mm	43.7±9.2	45.1±8.9	44.3±9.1	NS
First heart rate, bpm	119.23±33.4	118.9±31.1	119.0±32.3	.024
First SBP, mmHg	129.6±25.9	131.2±27.2	130.4±26.5	NS
First DBP, mmHg	79.7±15.8	78.4±16.6	79.0±16.2	<.001
Serum creatinine, μmol/L	110.0±96.7	97.4±84.2	104.1±91.3	NS
**Substances use**				
Tobacco use	398(37.4)	67(6.8)	465(22.7)	<.001
Alcohol consumption (n = 161)				<.001
Heavy drinker	8 (0.7)	1 (0.1)	9 (0.4)	
Moderate drinker	25 (2.3)	1 (0.1)	26 (1.2)	
Occasional drinker	66 (6.2)	8 (0.8)	74 (3.6)	
Former drinker	45 (4.2)	7 (0.7)	52 (2.5)	
Khat chewing (only in Yemen)	139 (13.0)	140 (14.2)	279 (13.6)	<.001
**Comorbidities**				
Coronary Artery diseases	318 (29.9)	258(26.3)	576(28.1)	.012
Hypertension	503(47.3)	570(58.1)	1073(52.5)	<.001
Hyperlipidemia	324(30.4)	356(36.3)	680(33.2)	<.001
Diabetes mellitus	289(27.1)	317(32.3)	606(29.6)	<.001
Left ventricular systolic dysfunction	231(21.7)	138(14.0)	369(18.0)	<.001
Pericarditis	5(0.47)	3(0.3)	8(0.4)	.026
Congenital heart disease	7(0.6)	7(0.7)	14(0.6)	NS
Rheumatoid heart disease	107(10.0)	211(21.5)	318(15.5)	<.001
Valvular heart disease	188(17.6)	206(21.0)	494(24.1)	<.001
**Past history**				
Transient ischemic attack	30 (2.8)	48 (4.9)	78 (3.8)	<.001
Stroke	91 (8.5)	95 (9.6)	186 (9.1)	NS
Hemorrhagic	9 (0.8)	7 (0.7)	16 (0.7)	
Ischemic	69 (6.5)	81 (8.2)	150 (7.3)	
Unknown	13 (1.2)	7 (0.7)	20 (0.9)	
Dementia/cognitive defects	50 (4.7)	33 (3.3)	83 (4.0)	.004
Sleep Apnea	12 (1.1)	12 (1.2)	24 (1.1)	NS
COPD (emphysema)	36 (3.3)	68 (6.9)	104 (5.0)	<.001
Dialysis	17 (1.6)	14 (1.4)	31 (1.5)	NS
Thyroid disease	28 (2.6)	75 (7.6)	103 (5.0)	<.001
Major bleeding	25 (2.3)	35 (3.5)	60 (2.9)	<.001
Type of Atrial fibrillation				NS
First attack ever	462 (43.5)	293 (29.9)	755 (37.0)	
Paroxysmal	182 (16.9)	171 (17.4)	351 (17.2)	
Permanent	287 (27.0)	389 (39.7)	676 (33.1)	
Persistent	98 (9.2)	95 (9.7)	193 (9.4)	
Unknown	36 (3.4)	32 (3.3)	68 (3.3)	
CHADS_2_ score	1.3±1.3	1.6±1.3	1.4±1.3	NS
0	355 (33.4)	227 (23.2)	582 (28.5)	
1	286 (26.9)	275 (28.1)	561 (27.5)	
≥2	422 (39.7)	478 (48.7)	900 (44.0)	
Reasons for ER visit				NS
Atrial fibrillation	516 (48.5)	407 (41.5)	923 (45.2)	
Cardiac	301 (28.3)	277 (28.3)	578 (28.3)	
Non-cardiac	246 (23.1)	296 (30.2)	542 (26.5)	

NS- not significant, UAE- United Arab emirates, COPD- chronic obstructive pulmonary diseases.

*Data are expressed as n(%) or mean± standard deviation

^†^Data of body mass index was missing in 121 patients ((male-1008, female-914; n = 1,922),

^‡^Data of LA size diameter was missing in 586 patients (n = 1456), CHADS_2_- Congestive heart failure, hypertension, age, diabetes mellitus, stroke/TIA_2_.

Regarding the overall treatment management (Table 2), men underwent pharmacological cardioversion more often (14.4% vs 8.2%), and women received digoxin more often than men (25.6% vs 17.4%). A total of 624 patients with AF were followed at the end of 1, 6, and 12 months for INR, a higher proportion of the women were followed with a mean INR levels of 2.5± 0.8 on 12-month follow-up. Majority of the women were discharged with AF rhythms (41.1%), whereas men with sinus rhythms (41.4%).

**Table 2:** Comparison of difference in treatment management between sexes.

Rhythm management	Men (n = 1063, %)	Women (n = 980, %)	Total (n = 2043, %)	P
Cardioversion	178 (16.7)	92 (9.3)	270 (13.2)	<.001
Cardioversion type (n = 270)				NS
Electrical	24 (2.2)	11 (1.1)	35 (1.7)	
Pharmacological	154 (14.4)	81 (8.2)	235 (11.5)	
Type of pharmacological agents (n = 154)				NS
Amidarone	95 (4.7)	64 (3.1)	159 (7.8)	
Amidarone + Propafenone	1	-	1	
Propafenone	53 (2.6)	11 (0.5)	64 (3.1)	
Ibutilide	1	3 (0.1)	4 (0.2)	
Flecainide	4 (0.2)	3 (0.1)	7 (0.3)	
Reasons for not considering cardioversion				.031
Rate control	672 (63.2)	724 (73.8)	1396 (68.3)	
Spontaneous cardioversion	106 (9.9)	78 (7.9)	184 (9.0)	
Others	107 (10.0)	86 (8.7)	193 (9.4)	
Other pharmacological drugs^†^				<.001
Diuretics	369 (34.7)	402 (41.0)	771 (37.7)	
ACE inhibitors	312 (29.3)	277 (28.2)	589 (28.8)	
ARBs	108 (10.1)	166 (17.0)	274 (13.4)	
Calcium channel blockers	114 (10.7)	161 (16.4)	275 (13.4)	
Beta-blockers	355 (33.3)	371 (37.8)	726 (35.5)	
Digoxin	185 (17.4)	251 (25.6)	436 (21.3)	
Aspirin	414 (38.9)	405 (41.3)	819 (40.1)	
Clopidogrel	85 (8.0)	74 (7.5)	159 (7.8)	
Statins	314 (29.5)	353 (36.0)	669 (32.7)	
Other lipid-lowering agents	20 (1.8)	11 (1.1)	31 (1.5)	
Warfarin	267 (25.1)	361 (36.8)	628 (30.7)	
LMWH	8 (0.7)	8 (0.8)	16 (0.8)	
Unfractionated heparin	3 (0.3)	4 (0.4)	7 (0.3)	
INR monitored				NS
Follow-up 1 INR (n = 624)	290 (27.2)	334 (34.0)	2.5±0.8	
Follow-up 6^th^ month INR (n = 560)	259 (24.3)	301 (30.7)	2.5±1.0	
Follow-up 12^th^ month INR (n = 547)	249 (23.4)	298 (30.4)	2.5±0.8	
Discharged with rhythms	849 (79.8)	763 (77.8)	1612 (79.0)	.015
AF rhythms	374 (35.1)	403 (41.1)	777 (38.0)	
Sinus rhythms	440 (41.4)	319 (32.5)	759 (37.2)	
Inapplicable (died)	35 (3.3)	41 (4.2)	76 (3.7)	

NS- not significant,

^†^Some patients took more than one drug.

Of 1776 patients with AF who received antithrombotic and anticoagulants during follow-up, a significant number of women with CHADS_2_-VASc score >2 received warfarin (31% vs 8.7%), warfarin with aspirin (7.2% vs 5.9%) and antiplatelet therapy (2.7% vs1.9%) during the hospital stay (Table 3). After following 2043 patients for one year, all causes of mortality rate was 15%, stroke (6.4%), and major bleeding events (1.7%) with no significant differences between men and women. However, recurrent ER visit, hospital admission for AF and HF was more often in women than men (Table 4).

**Table 3:** Comparison of differences in CHADS_2_ with warfarin and antiplatelet therapy between sexes (n = 1776).

CHADS2-VASc	0 (n = 425, %)		1–2 (n = 234, %)		>2 (n = 1117, %)		*P*
	Men	Women	Men	Women	Men	Women	-
Warfarin alone	74 (4.1)	-	78 (4.4)	74 (4.1)	156 (8.7)	553 (31.1)	<.001
Dual therapy	11 (0.6)	-	27 (1.5)	7 (0.4)	106 (5.9)	128 (7.2)	.001
Triple therapy	1	-	1	-	19 (1.0)	15 (0.8)	.002
Antiplatelet	288 (16.2)	-	11 (0.6)	3 (0.1)	35 (1.9)	49 (2.7)	NS
None	51 (2.8)	-	16 (0.9)	17 (0.9)	22 (1.2)	34 (1.9)	.030

**Key**: CHADS_2_: Congestive heart failure, Hypertension, Age ≥75 years, Diabetes mellitus, Stroke/TIA_2_. Dual therapy: warfarin and either of the two antiplatelets (aspirin or clopidogrel). Triple therapy: warfarin plus aspirin plus clopidogrel. Antiplatelet: aspirin or clopidogrel or both. None: neither warfarin nor antiplatelet. NS: not significant, 1784 completed the follow-up.

**Table 4:** Sex differences in outcome events at 12 months follow-up in the entire cohort according to the reasons for emergency room visit (N = 2043).

	Gender		Reasons for ER visit					
*Events*	*Male (52%)*	*Female (48%)*	*Atrial fibrillation (n = 705)*		*Cardiac (n = 510)*		*Non-cardiac (n = 436)*	
			Male	Female	Male	Female	Male	Female
All cause death	155	151	22	23	64	46	69	82
Stroke/TIA	64	68	12	14	25	17	27	37
Peripheral embolization	3	2	0	0	0	1	3	1
Major bleeding	17	19	3	6	8	2	17	11
*Gastrointestinal*	8	4	1	1	3	0	4	3
*Intracerebral*	3	0	0	0	2	0	1	0
*Subdural*	1	1	0	0	1	0	0	1
*Others*	1	1	0	0	1	0	0	1
Recurrent ER visit for AF	128	144	71	85	38	36	19	23
Recurrent hospital admission for AF	103	106	56	55	34	29	13	22
Recurrent hospital admission for HF	116	120	28	38	61	53	27	29

TIA-transient ischemic attack, ER- emergency room, AF- atrial fibrillation, Apart from all-cause mortality and stroke/TIA.

Significant risk factors were noticed between the sexes when the patients were stratified by age (>65 years), smoking, alcohol use and CHADS_2_ scores. For instance, men with CHADS_2_ score ≥5 showed thrice higher risk for atrial fibrillation than women. Similarly, social habits such as moderate alcohol drinking and smoking showed higher odds in men than women (Table 5).

**Table 5:** Comparison of atrial fibrillation risk factors between sexes.

Risk factors	Atrial fibrillation rate (95% CI)	
	Male	Female
Age ≥65 years	1.4 (1.2–1.7)	0.6 (0.5–0.8)
Smoking	8.2 (6.2–10.8)	4.1 (1.7–10.0)
Alcohol use (moderate drinkers)	26.2 (3.5–193.7)	3.1 (0.1–55.8)
Coronary artery disease	1.2 (0.9–1.4)	1.3 (0.5–3.0)
Left ventricular systolic dysfunction	1.7 (1.3–2.1)	1.0 (0.6–1.8)
History of heart failure	1.0 (0.9–1.3)	1.1 (0.3–4.0)
Cardiac surgery less than 30 days	1.1 (0.4–2.7)	3.0 (0.4–19.6)
ER visit (Atrial fibrillation)	1.5 (1.2–1.8)	0.6 (0.5–0.8)
CHADS_2_ score		
0	4.1 (1.0–15.8)	0.2 (0.6–0.9)
1	2.7 (0.7–10.5)	0.3 (0.09–1.3)
2	2.2 (0.5–8.4)	0.4 (0.1–1.7)
3	2.5 (0.6–9.6)	0.4 (0.1–1.5)
4	2.4 (0.6–9.6)	0.4 (0.1–1.6)
≥5	3.5 (0.8–15.0)	0.3 (0.06–1.2)

CI: confidence interval, CHADS_2_- Congestive heart failure, Hypertension, Age ≥75 years, Diabetes mellitus, Stroke/TIA_2_.

## Discussion

The present multinational investigation provides evidence for differences between male and female in the management and outcomes of AF during one year follow up in Gulf region of the Middle East. Thus, patients with AF in Arab countries of the Middle East were relatively higher in men (mean age 55.1±16.5) than women (58.5±16.2). These results were consistent with recent meta-analysis conducted on 13 prospective studies which examined the risk of AF in men age <50 years (RR: 1.58, 95% CI: 1.28–1.95) is associated with increased physical activity exposure [10]. These differences could be due to low physical activities in Arab population as a favorably modified risk factor in men with AF. However, advancing age is considered as the most important risk factor that can double AF incidence with every 10-year increase in age [11]. In our study, nearly forty percent of the women were aged ≥65 years, compared to 31% of men.

Several epidemiological studies worldwide have described elevated BMI, hypertension, diabetes mellitus, coronary artery disease, valvular heart disease and heart failure were major risk factors that vary between women and men [11–13]. Similar findings were also noticed in our cohort where significant differences were noticed between men and women with AF. For instance, in our study, women showed higher BMI than men with AF (28.6±7.2 kg/m^2^ vs. 27.3±5.3 kg/m^2^; *p*<0.001). In our study, women with AF have higher prevalence of hypertension and valvular heart disease than men and these findings were consistent with other epidemiological studies [11, 14–15]. Several studies evaluated genetic and hereditary factors that predispose to AF. However, future research should stimulate to investigate these risk factors in our population to AF.

The American College of Chest Physicians did not include female sex in the calculation of stroke risk (CHADS_2_)—based treatment recommendations [16]. Similarly, Canadian Cardiovascular Society and the Japanese Cardiovascular Society also recommended the use of the same model [17,18]. In our study, nearly half of the women (48.7%) had higher CHADS2 scores (≥2) which suggests a higher risk factor for stroke in women than men. Similarly in 2012, *Friberg et al*. study found a higher risk of stroke in females of all ages; but significant higher risk was noticed in women aged ≥75 years (hazard ratio 1.23; *p*<0.001) than men [19]. Contradictorily, *Olesen et al*. study identified females <65 years had low stroke risk than males. In addition, this study also identified that this age group of females carries a higher risk of hospital admission and death due to thromboembolism than males [20]. However, it is notable that data in stroke risk of females aged 65–74 years still remains conflicting. Further investigations are needed to better clarify this aspect.

Although the pathophysiology of AF has been extensively investigated over the years, the differences between men and women cardiac anatomy and electrical mechanism contributing ER visit is still insufficient. In our study, a higher proportion of men visited ER with AF (48.5% vs 41.5%) and women with non-cardiac reasons (30.2% vs 23.1%). In a study of patients visiting the ER showed, women were more likely to have longer duration of atypical symptoms such as weakness and fatigue that contributing to worsen outcomes and quality of life in women [21]. However, these variations by sex differences has not been well reported.

Cardioversion to normal sinus rhythms is equally successful in men and women; however, women were less likely to undergo electric cardioversion than men [22]. Similar findings were noticed in our study, where only a negligible percent (1.1%) of women underwent electrical cardioversion than men (2.2%). But, a higher proportion of women were not considered for cardioversion due to rate control compared to men (73.8% vs 63.2%). Furthermore, women received digoxin treatment more often (25.6% vs 17.4%), statins (36% vs 29.5%) and warfarin therapy (36.8% vs 25.1%). These apparent differences in sexes were also noticed in Spanish population with different demographic characteristics and older aged women [23]. The higher use of digoxin and the lower rate of electrical cardioversion in women was more likely due to higher prevalence of persistent/permanent AF. Thus, these clinical entities considered less favorable condition for cardioversion.

Several reviews have examined stroke risk factors in AF patients focusing on sex differences in stroke risk. Numerous risk factors contribute to an individual’s risk of stroke in patients with AF. Many are well-established such as congestive heart failure, hypertension, advancing age (≥ 75 years), diabetes mellitus and a history of stroke or transient ischemic attack (TIA)—CHADS_2_ stroke risk scoring system [24]. More recent validated risk factors, such as vascular disease, age 65–74 years and female gender (CHADS_2_-VAS) may also be associated with stroke risk in AF patients [25]. In our study, nearly one-third of the female AF patients under warfarin medication had higher thromboembolic stroke risk (CHADS2-VAS ≥2) than men (31.1% vs 8.7%). This striking differences in CHADS2-VASc scores between men and women groups suggests that women visiting ER at later stage may develop higher thromboembolic risk. Thus, this may be due to higher prevalence of comorbidities such as diabetes, hypertension, high BMI and poor physical activities in women. A 2011 review carried out by Women’s Health identified similar risk factors in women but does not highlight female AF patients with greater number of co-morbidities which increase the stroke risk in women than men [26].

During one-year follow-up, high mortality rate (15%) was noticed in our patients with no difference in their sexes. However, lower mortality rate was noticed in AF patients (2.2%) and highest (4%) noticed in women patients presenting with non-cardiac reasons like stroke, infection or respiratory diseases. This mortality could be related to their comorbidities associated with AF in women patients.

Our study on gender differences highlights key differences in men and women with AF from the Middle Eastern region. It is important to recognize the significant differences in management and outcomes between the sexes. It is striking that women received more conservative treatment despite having greater comorbidities. In fact, women received digoxin to control the heart rate more often than men and had fewer indication for electrical cardioversion. Greater age, higher BMI, Khat chewing (in Yemen), comorbidities, with TIA’s and with major bleeding events were some of the most significant factors for women being referred to a cardiologist. Higher rates of smoking, alcohol use, and CHADS2 scores were some of the significant risk factors in men with AF. Through our investigation it suggests a slightly higher risk of complications with AF ablation noticed in women. This reflects there is a need for novel modalities to improve and management of AF might be important area in the future.

## Study strengths and limitations

Our study is a prospective design, with high follow-up rate (96%) and leading validity. The near equal representation of both genders allowed us to make our findings representative of the region. However, this study is not without limitations. Firstly, we enrolled only patients with AF who presented to the ERs in the hospital. We did not include the patient’s data who were not enrolled and the reasons for not enrolling. Secondly, this was a sub-group study that was not specially designed to analyze sex differences. Lastly, the number of events identified were low and thus some estimations could be reliable. This sub-group study helped us to reflect the actual practice at different care levels at different health centers attributable to the inclusion method.

## Conclusion

Gender differences observed in the management and outcomes of AF in Middle Eastern region are relatively noticed in older women with high burden of atherosclerotic risk factors. Suboptimal use of anticoagulants, higher mortality and stroke/TIA events at one year are high but similar between the sexes. ER management revealed high use of rate control strategy and high rate of hospital admission in women, which need newer modalities to improve management and outcomes and also to reduce the cost of management.

## Supporting information

S1 File List of Gulf SAFE investigators. (DOC)
