# Supplementary material for: Sex differences in management and outcomes of patients with atrial fibrillation in the Middle East: Gulf survey of atrial fibrillation events (Gulf SAFE)
Source: PLoS One. 2017 May 17;12(5):e0175405. doi: 10.1371/journal.pone.0175405 (PMC5435140; doi:10.1371/journal.pone.0175405)
Supplement: S1 File — (DOC) [file pone.0175405.s001.doc]

**Appendix**

**Gulf SAFE Administrative Organization**

**Steering Committee**

Mohammad Zubaid (chair and principal investigator), Wafa Rashed (registry coordinator), Wael Al-Mahmeed and Abdulla Shehab (UAE), Kadhim Sulaiman (Oman), Nidal Asaad (Qatar), Haitham Ameen (Bahrain), Ahmed Al-Qudaimi (Yemen), Alawi A. Alsheikh–Ali (UAE), Ibrahim Al-Zakwani (biostatistician).

**National Coordinators (NC) and Investigators**

**Bahrain:** Haitham Ameen (NC); Adel K. Hamad*, Rajesh Jayakumar, Babu Thevan, Devashish Sheel, Manal Matar, Hugmi B. Jain, Fawaz Bardooli, Wala Mattar.

**Kuwait:** Wafa Rashed (NC); Adan Hospital: Mustafa M. Ridha*, Amin S. Amin, Bobby Cherian; Mubarak AlKabeer Hospital: Hisham Saad*, Ashraf Hamad, Hani Abdul Salam, Maged Yousef, Ihab Awwad, Gladis Hefny, Nabil Ragab, Mohammad Abdul Moneim, Sameh El Masry, Mohammad Samir; Farwaniyah Hospital: Fahad Al-Enezi*, Ashraf Abdelwahed, Mahmoud Abdultawab, Musaad Elbanna, Ayman Abdulmogoud.

**Oman:** Kadhim Sulaiman (NC); Sohar Hospital: Marei Aysha*, Ali Alkhafaji, Mohammad Agar, Hamada M. Amin, Hatem H. Mohammed, Abu Baker, Nashat Abdulhalim, Pasupathy Seshadri, Sohail Shahzad; Sultan Qaboos Hospital: Prit P. Singh*, S. Wettewe; Royal Hospital: Najeeb Al-Rawahi*, Hood Al-Abri; Nizwa Hospital: Faisal Al Tamimi*, Dilip Kumar, Saquib Ahmed, Refaat Abdulla, Ahmed Hagazy, Abdulslam Abdulrahman, M.R. Shajee, Abu Baker Elsadiq, Ahmed Moawed, Wael Mohammed, Abdulwaheed Baig; Ibri Hospital: Sharat K. 23 Samantray*, Mohammed N. El-Nour, Waseem Mohammad; Ibra Hospital: Narayan A. Narayan*, Elmutaz Elamin, Addulnasser Awadh, Sharath Ali.

**Qatar:** Nidal Assad(NC), Soaly E*, Ahmad Shaaban, Esam Eljerjawy, R.F. Al-Aqeedi, Gomaa M. Maauf.

**United Arab Emirates**: Abdulla Shehab (NC); Al-Noor hospital (Al-Ain): Jasim AlHayali*, Eman Mohammed; Dubai Hospital: Dr Afzalhussein Yusufali*, Ambreen F. Tariq; Fujairah Hospital: Amrish Agrawal*, Abdulrahman Aboobaker; Ras Al Khaima Hospital: Adel Wassef*, Ajaz alhaq; Rashed Hospital (Dubai): Fahad Baslaib*, Ali Raza, Sameera Ali, Kabad Rao; Shaikh Khalif Medical City (Abu Dhabi): Wael Almahmeed*, Irfan Maqsood, Amer Sana; Zayed Military Hospital (Abu Dhabi): Salim AlKaabi*, Waheed Murad.

**Yemen:** Ahmed Al-Qudaimi (NC); Al-Thawra Hospital (Sanna’a): Abdulwahab Almatry*, Sameera Al-Ragwi*, Ali Othman, Afrah Almulsi, Mohammed Alshami; Al Jamhuri Teaching Hospital (Sana’a):Abdu Hamod*; Modern Medical Center (Aldalee): Motea Alawlaqi*, Moaeen Hussein; Al-Wehdah Teaching Hospital: Fuad Ali*; Al-Jamhuria Hospital (Aden): Salem Almehdar*.

*Chief site officer.
